# MR-GGI: accurate inference of gene–gene interactions using Mendelian randomization

**DOI:** 10.1186/s12859-024-05808-4

**Published:** 2024-05-15

**Authors:** Wonseok Oh, Junghyun Jung, Jong Wha J. Joo

**Affiliations:** 1 Department of Industrial Pharmacy, Dongguk University-Seoul, Seoul, 04620 South Korea; 2 Department of Computational Biomedicine, Cedars-Sinai Medical Center, Hollywood, CA USA; 3 Department of Computer Science and Engineering, Dongguk University-Seoul, Seoul, 04620 South Korea; 4 Division of AI Software Convergence, Dongguk University-Seoul, Seoul, 04620 South Korea

**Keywords:** Gene–gene interactions, Mendelian randomization, Gene regulatory network, Yeast GRN

## Abstract

**Background:**

Researchers have long studied the regulatory processes of genes to uncover their functions. Gene regulatory network analysis is one of the popular approaches for understanding these processes, requiring accurate identification of interactions among the genes to establish the gene regulatory network. Advances in genome-wide association studies and expression quantitative trait loci studies have led to a wealth of genomic data, facilitating more accurate inference of gene–gene interactions. However, unknown confounding factors may influence these interactions, making their interpretation complicated. Mendelian randomization (MR) has emerged as a valuable tool for causal inference in genetics, addressing confounding effects by estimating causal relationships using instrumental variables. In this paper, we propose a new statistical method, MR-GGI, for accurately inferring gene–gene interactions using Mendelian randomization.

**Results:**

MR-GGI applies one gene as the exposure and another as the outcome, using causal cis-single-nucleotide polymorphisms as instrumental variables in the inverse-variance weighted MR model. Through simulations, we have demonstrated MR-GGI's ability to control type 1 error and maintain statistical power despite confounding effects. MR-GGI performed the best when compared to other methods using the F1 score on the DREAM5 dataset. Additionally, when applied to yeast genomic data, MR-GGI successfully identified six clusters. Through gene ontology analysis, we have confirmed that each cluster in our study performs distinct functional roles by gathering genes with specific functions.

**Conclusion:**

These findings demonstrate that MR-GGI accurately inferences gene–gene interactions despite the confounding effects in real biological environments.

**Supplementary Information:**

The online version contains supplementary material available at 10.1186/s12859-024-05808-4.

## Background

For decades, many studies have focused their efforts on identifying the regulatory processes between genes to uncover their potential functions. For example, Shi et al. reviewed the role of *Oct4* performing various regulating function [1, 2]. The studies have revealed that some of the genes encode several transcription factors (TFs) or protein hormones to regulate the expression of other genes. Gene regulatory network (GRN) analysis is one of the most popular approaches for uncovering these regulatory processes. Genes within the GRN are connected through regulatory relationships; thus, identifying the gene–gene interaction is essential for constructing the GRN. GRN inference studies utilize various gene–gene interaction algorithms, for which identifying the accurate direction of gene–gene interaction is important.

With the advancement of genome-wide association studies (GWAS) [3–5] and expression quantitative trait loci (eQTL) studies [6–8], the amount of genomic data has increased dramatically, making it possible to infer gene–gene interactions more accurately than ever. It is well known that there are various unknown confounding factors that distort gene–gene interactions and make their relationship ambiguous. Mendelian randomization (MR) is an emerging tool for causal inference analysis in genetics, as it successfully infers causality while overcoming problems of confounding effects. The MR model is designed to estimate the causal effects of an exposure (i.e., gene) on an outcome (i.e., trait) by leveraging an instrumental variable (IV) such as genetic variants, which adjusts bias caused by confounding effects. Two-sample MR [9] is one of the foundational MR models, and there are several other MR models such as inverse-variance weighted (IVW) MR [10], MR-Egger [11], MR weighted median [12], and multivariable MR [13] that are developed based on foundational MR principles to enhance the precision of causal inference.

In this paper, we propose a new statistical method referred to as “MR-based method for inferring Gene–Gene Interaction (MR-GGI),” which accurately infers interactions between genes utilizing the MR. MR-GGI infers relationships between two genes by applying one gene as the exposure, the other gene as the outcome, and one or more causal cis-SNPs for the genes as the IV(s) in the IVW MR model [10]. Utilizing various simulated datasets, we show that MR-GGI successfully controls the type 1 error and retains its statistical power even though confounding effects exist in the data. In addition, we show that using more than one cis-SNP as IVs increases the statistical power of experiments in simulation studies. Utilizing the DREAM5 dataset [14], which is often used as a gold standard dataset for GRN studies, we show that MR-GGI accurately infers gene–gene interactions and results in a superior F1 score compared to existing methods that are designed to infer biological networks. Lastly, to demonstrate that MR-GGI works successfully on data in a real biological system, we construct a yeast GRN with six clusters utilizing a yeast dataset [15]. By performing a functional enrichment analysis with Gene Ontology, we found that three of the clusters are involved in cytoplasmic gene expression and one independent cluster is mainly involved in mitochondrial translation.

## Results

### Overview of MR-GGI

It is important to accurately infer gene–gene interactions for uncovering potential functions in a GRN. However, genomics studies are often challenged by various unknown confounding factors that influence gene–gene interactions and lead to misinterpretations. MR is one of the most popular tools for causal inference as it adjusts bias induced by confounding effects. MR incorporates an IV in the model to infer the causality between exposure and outcome. We propose a new statistical method, MR-GGI, which utilizes MR to accurately infer gene–gene interactions. MR-GGI identifies gene–gene interaction by inferring causality between two genes, where one gene is used as an exposure, the other gene is used as an outcome, and causal cis-SNP(s) for the genes are used as IV(s).

**Fig. 1:**
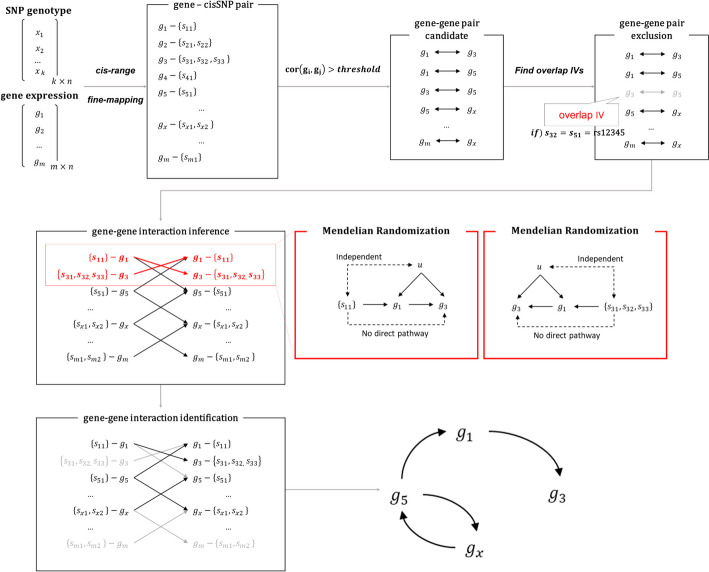
Overview of MR-GGI. MR-GGI tests the causal relation between two genes using MR. ${g}_{i}$ represents gene $i$, ${s}_{ij}$ represents $j$th cis-SNP for ${g}_{i}$, and $u$ represents an unknown confounding factor that affects genes. The red box shows an example of causality test on ${g}_{1}$ and ${g}_{3}$ utilizing MR, where ${s}_{11}$ represents a cis-SNP for ${g}_{1}$ and ${s}_{31}$, ${s}_{32}$, ${s}_{33}$ represent three cis-SNPs for ${g}_{3}$ that were found after the fine-mapping and filtering processes

Figure 1 shows an overview of MR-GGI. MR-GGI requires gene expression and the genotype of the data. We apply a fine-mapping method, such as sum of single effects linear regression (susieR [16, 17]) to identify a set of cis-SNPs consisting of independent variants for each gene. These cis-SNPs are subsequently utilized as IVs. A threshold is used to find gene–gene interaction pair candidates that are to be tested. From which, pairs with overlapping cis-SNPs that effect both exposure gene and outcome gene are excluded considering the pleiotropic effects. Utilizing the cis-SNP sets and gene–gene pair candidates, MR-GGI infers gene–gene interactions based on the IVW MR model [10]. Let’s say we are testing the interaction between two genes, ${g}_{1}$ and ${g}_{3}$, and ${s}_{1}=\{{s}_{11}\}$ and ${s}_{3}=\{{s}_{31},{s}_{32},{s}_{33}\}$ are the sets of cis-SNPs for ${g}_{1}$ and ${g}_{3}$, respectively. MR-GGI infers the causal relationship between ${g}_{1}$ and ${g}_{3}$, assuming 4 scenarios: ${g}_{1}$ affects ${g}_{3}$; ${g}_{3}$ affects ${g}_{1}$; ${g}_{1}$ and ${g}_{3}$ affect each other; and ${g}_{1}$ and ${g}_{3}$ are independent. To test whether ${g}_{1}$ affects ${g}_{3}$, MR-GGI applies MR using ${g}_{1}$ as exposure, ${g}_{3}$ as outcome, and ${s}_{1}$ as IV. To test whether ${g}_{3}$ affects ${g}_{1}$, MR-GGI applies MR using ${g}_{3}$ as exposure, ${g}_{1}$ as outcome, and ${s}_{3}$ as IV. If both tests apply, we say that ${g}_{1}$ and ${g}_{3}$ affects each other. If none of the tests apply, we say that ${g}_{1}$ and ${g}_{3}$ are independent of each other.

### MR-GGI controls type I errors in simulation studies

**Fig. 2:**
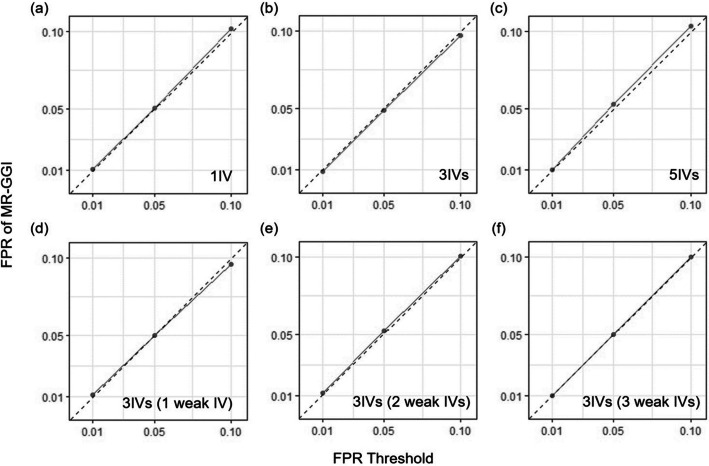
False positive rate (FPR) of MR-GGI in simulation studies. The X-axis represents the FPR threshold, and the Y-axis represents the FPR of MR-GGI. **a**, **b**, and **c** show the results of simulated data, where 1, 3, and 5 cis-SNPs are used as IV, respectively. **d**, **e**, and **f** show the results of simulated data where 3 cis-SNPs are used as IV; among them, 1, 3, and 5 cis-SNPs have weak effect size of 0.1, respectively

Simulated datasets were generated based on a previous MR model [18], which allows multiple instrumental variables (see “Material and methods”). To show that MR-GGI controls type I errors in various scenarios, we simulated various datasets with different options. First, to show that MR-GGI controls the false positives in cases of different numbers of IVs, we simulated 3 sets of 10,000 datasets with two genes. Each gene has no effect on the other, and consists of either 1, 3, or 5 cis-SNPs with effect sizes in the range of 0.25–0.6 (see “Material and methods”). For different thresholds of 0.01, 0.05, and 0.1, MR-GGI successfully controls false positives regardless of the number of cis-SNPs used in the model (Fig. 2a–c).

Second, we investigated the case when datasets contain IV with a weak effect size. Here, we define a weak IV as a IV with small effect size of 0.1, following a previous study [18]. We simulated 3 sets of 10,000 datasets of two genes with no effects on each other. Each gene consists of 3 IVs, which contain either 1, 2, or 3 weak IVs out of 3 IVs. The results show that MR-GGI successfully controls false positives and that it is robust to either the number or effect sizes of IVs in the MR model (Fig. 2d–f). In addition, we investigated the case when the variance of the effect size estimate is large as IV could be weak in the case even though the effect size estimate is large. As a result, we observed that MR-GGI successfully controlled type I error in the case (data not shown).

Third, we simulated data with confounding effects to show that MR-GGI successfully controls false positives under confounding effects. We simulated 10,000 datasets with two genes and no effects on each other. Each gene consists of 3 IVs. The genes are correlated to each other due to confounding effects (*ρ*), which is in the range of − 0.9 to 0.9 (see “Material and methods”). Table 1 shows that MR-GGI robustly controls false positives.

**Table 1:** False positive rate (FPR) of MR-GGI under 7 different values of confounding effects ($\rho$)

FPR	Correlation of ${u}_{1}$ and ${u}_{2}$ ($\rho$)						
	− 0.9	− 0.5	− 0.2	0	0.2	0.5	0.9
*Cut-off*							
0.1	0.1026	0.1041	0.1043	0.0990	0.1015	0.1031	0.1048
0.05	0.0517	0.0528	0.0521	0.0509	0.0523	0.0520	0.0560
0.01	0.0113	0.0108	0.0107	0.0111	0.0109	0.0106	0.0119

### MR-GGI retains statistical power under confounding effects in simulation studies

**Fig. 3:**
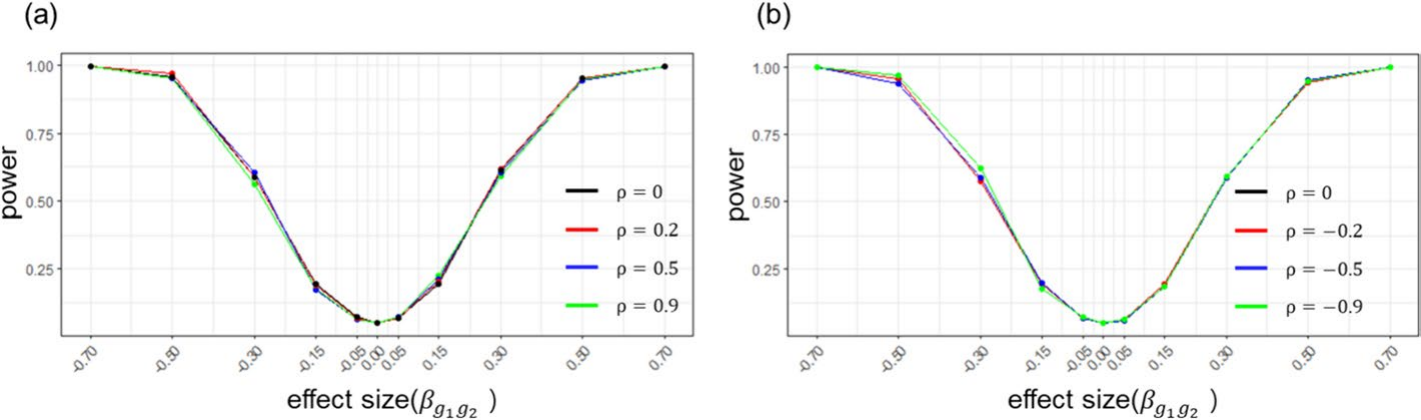
The power curve of MR-GGI for datasets with different confounding effects ($\rho$). The X-axis represents the effect size estimate of ${g}_{1}$ to ${g}_{2}$(${\beta }_{{g}_{1}{g}_{2}}$), and the Y-axis represents the statistical power. **a** The plot shows power curves for positive $\rho$. The black, red, blue, and green lines show the power curves for $\rho$ at 0, 0.2, 0.5, and 0.9, respectively. **b** The plot shows power curves for negative $\rho$. The black, red, blue, and green lines show the power curves for $\rho$ at 0, − 0.2, − 0.5, and − 0.9, respectively

To show that MR-GGI retains statistical power under confounding effects, we conducted simulation analysis using simulated data with various confounding effects. We simulated datasets with two genes; for each gene, 3 cis-SNPs were simulated and used as IVs in the model. To test the power in various cases, we simulated datasets for which ${g}_{1}$ has effect on ${g}_{2}$ with different effect sizes (${\beta }_{{g}_{1}{g}_{2}}$) in the range of − 0.7 to 0.7 and different correlation sizes due to the confounding effects ($\rho$) in the range of − 0.9 to 0.9. A total of 1000 simulated datasets were used for each case (see “Material and methods”). Figure 3 shows the power curves with different ${\beta }_{{g}_{1}{g}_{2}}$ and $\rho$. The results show that MR-GGI successfully retains statistical powers under various confounding effects.

**Fig. 4:**
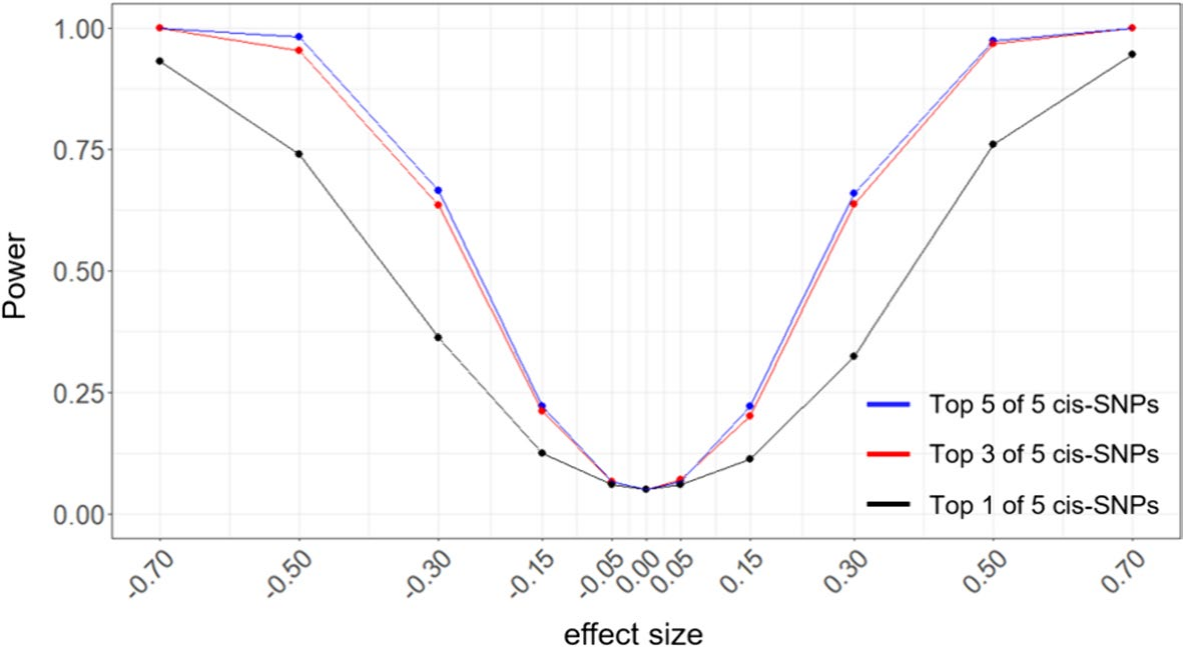
The power curve for different number of IV(s) used in the model. The X-axis represents the effect size estimate (${\beta }_{{g}_{1}{g}_{2}}$), and the Y-axis represents the statistical power. The blue, red, and black lines show the power curve when the top 5, 3, and 1 cis-SNPs are used as IVs in the model, respectively

In addition, we evaluated the statistical power of MR-GGI using different numbers of causal cis-SNPs in the MR model as IVs. We simulated datasets with two genes, ${g}_{1}$ and ${g}_{2}$, where ${g}_{1}$ has an effect on ${g}_{2}$ with an effect size (${\beta }_{{g}_{1}{g}_{2}})$ in the range of − 0.7 to 0.7. A total of 5 cis-SNPs with effect sizes in the range of 0.25–0.6 were simulated for each gene. The result of MR-GGI, where 1, 3, and 5 cis-SNP(s) are used in the model as IVs to find gene–gene interactions (Fig. 4). The ones with the strongest effect size, referred to as top cis-SNPs, were selected as IV(s) among the 5 simulated cis-SNP(s), and 1000 datasets were simulated for each case (see “Material and methods”). The result shows that the statistical power increases with the number of IVs used in the model; especially, using more than 1 IV increases the statistical power significantly.

### MR-GGI accurately identifies gene–gene interactions in the DREAM5 dataset

We evaluated our method using the DREAM5 dataset [14], which is one of the gold standard datasets for testing gene–gene interactions based on IVs. We compared our method with existing causal inference methods: PC algorithms [19]; MR-based PC (MRPC) method [20]; and a Bayesian network with the Max–Min Hill Climbing (MMHC) method [21].

**Fig. 5:**
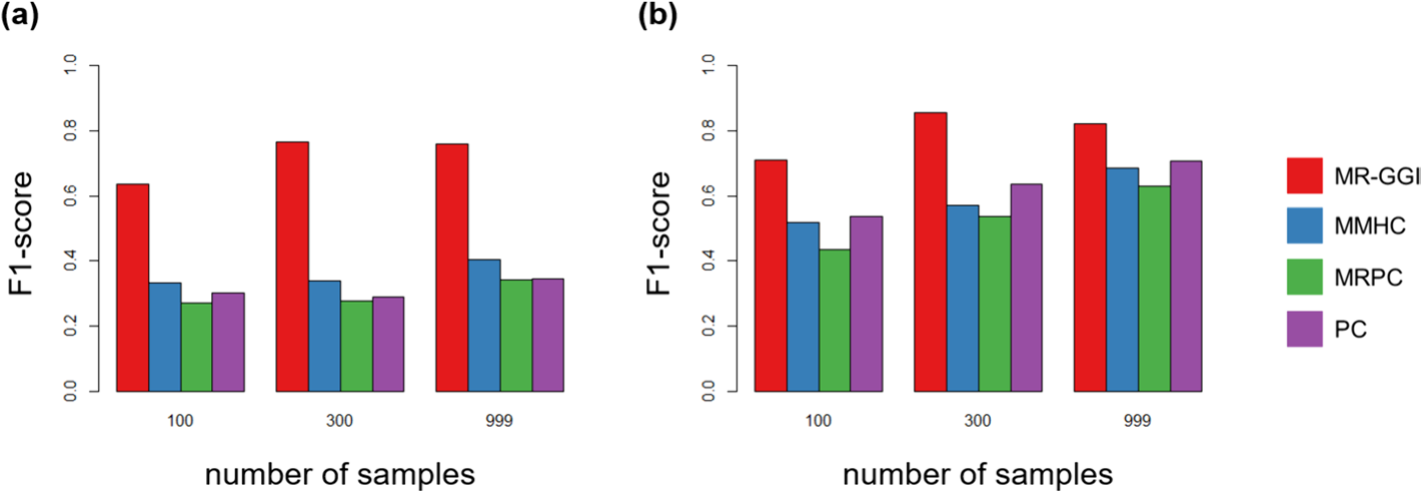
Comparison of MR-GGI with other methods using the DREAM5 dataset. MR-GGI, MMHC, MRPC, and PC methods were applied to the DREAM5 dataset with 100, 300, and 999 samples. Correlation between eGenes > 0.5 was used for the analysis. The bar graph shows the average F1-score of four different networks in the DREAM5 dataset. **a** The barplot shows the result when all genes with cis-SNPs reported by the DREAM5 dataset were used. **b** The barplot shows the result when weak IVs are filtered out of the dataset

We compared F1 scores applying each method to the DREAM5 dataset with 3 different sample sizes. Gene pairs with a correlation (${\beta }_{{g}_{i}{g}_{j}})$ of > 0.5 were used in the experiments. Figure 5a shows results when all the genes with cis-SNPs reported by the DREAM5 dataset were used in the experiment. Additionally, we compared F1 scores when weak IVs, which are cis-effect (${\beta }_{{s}_{i}{g}_{i}})$ < 0.2, were filtered out in the experiment. As a result, MR-GGI shows higher F1 scores in all sample sizes compared to other methods. Notably, the results show that when the data contains cis-SNPs with weak effect sizes, MR-GGI consistently demonstrates robust performance, while others do not.

### MR-GGI finds gene–gene interaction in the yeast dataset

**Fig. 6:**
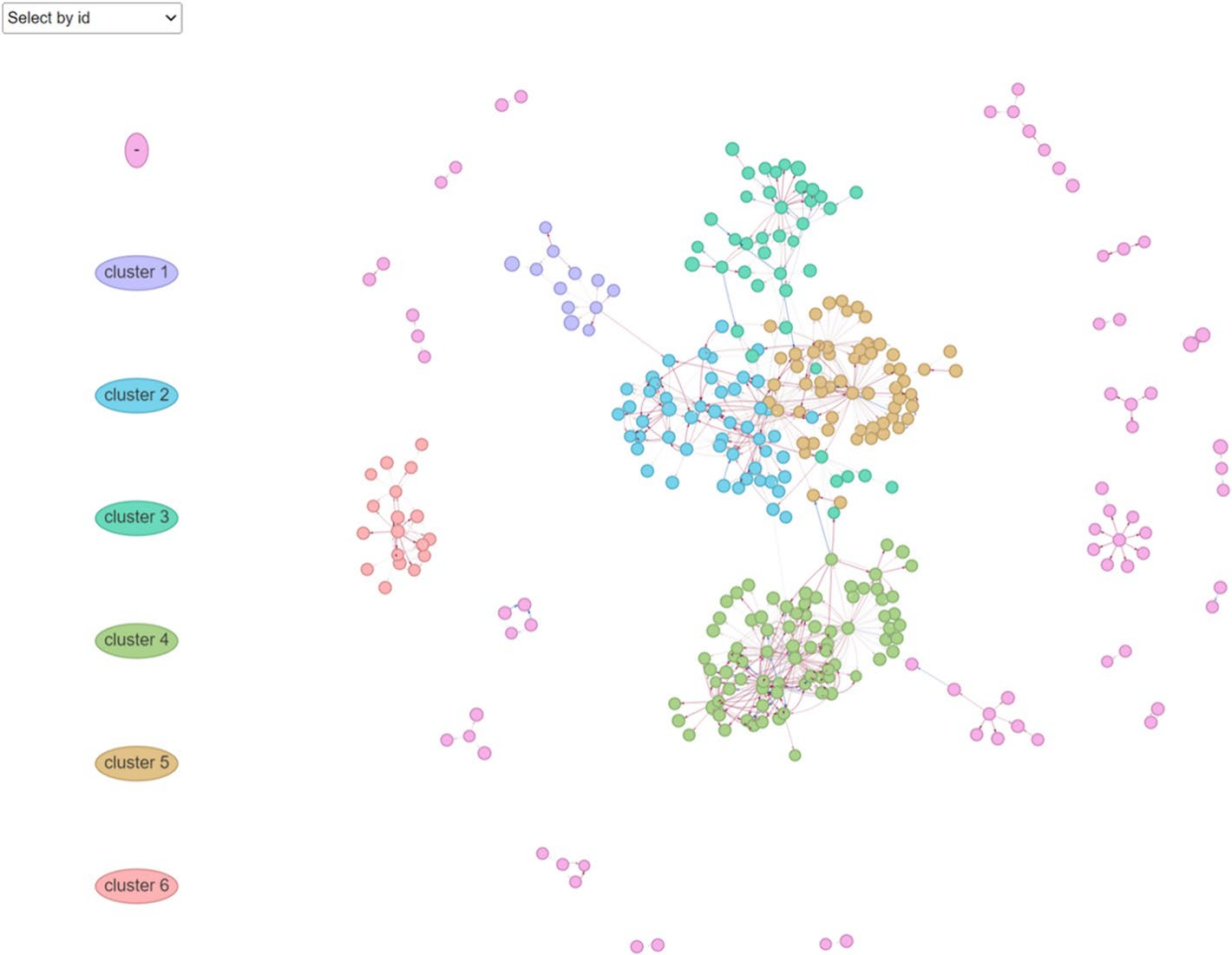
Yeast GRN with gene–gene interactions estimated from MR-GGI. Six clusters consisting of 331 nodes and 683 edges were found

We applied MR-GGI to a yeast dataset [15] to identify gene–gene interactions and construct a GRN using real datasets. MR-GGI found a total of 683 gene–gene interactions between 331 genes. Then we applied the Louvain clustering algorithm [22] to find six sub-network clusters (Fig. 6). Here, eGenes were filtered out using an absolute correlation of at least 0.75(see “Material and methods”). Next, to find the key role for each cluster, we calculated the degree of centrality for each gene in each cluster. We then identified biological process of Gene Ontology terms [23] by 2-step. In clusters 1, 2, 3, 4, 5, and 6, we found 14, 25, 15, 40, 3, and 8 GO BP terms (*p* < 0.05), respectively (Supplementary Data 1–6).

In cluster 3 (C3), cytoplasmic translation (GO:0002181), translation (GO:0006412), peptide metabolic processes (GO:0006518), peptide biosynthetic processes (GO:0043043), and gene expression (GO:0010467) were significantly enriched. In C4, ribosome biogenesis (GO:0042254), ribonucleoprotein complex biogenesis (GO:0022613), rRNA processing (GO:0006364), rRNA metabolic processes (GO:0016070), ribosomal large subunit biogenesis (GO:0042273), ribosomal small subunit biogenesis (GO:0042274), and gene expression (GO:0010467), were enriched. In C6, mitochondrial translation (GO:0032543), mitochondrial gene expression (GO:0140053), and translation (GO:0006412) were enriched. Notably, mitochondrial translation and mitochondrial gene expression were only enriched in C6. This result shows that C6 is involved in translation that occurs in ribosomes located in mitochondria differently from C3.

C2 is related to NADP and NADPH metabolism. We identified pyridine nucleotide metabolic process (GO:0019362), cellular response to oxidative stress (GO:0034599), pyridine-containing compound metabolic process (GO:0072524), response to oxidative stress (GO:0006979), NADPH regeneration (GO:0006740), and NADP metabolic process (GO:0006739). Response to desiccation (GO:0009269) and cellular response to desiccation (GO:0071465) were also identified in C2. Through this result, we predicted that NADP and NADPH metabolism system may be associated with desiccation/rehydration in yeast [24]. Additionally, C1 and C5 are the clusters related to ATP metabolism and regulation process (Table 2).

**Table 2:** Summary of yeast GRN constructed by MR-GGI

Cluster	Number of genes	After filtering out	Key role
1	12	3	ATP metabolism
2	51	17	NADP and NADPH metabolism
3	41	17	Cytoplasmic translation
4	80	40	Ribosome biogenesis
5	59	4	Regulation process
6	18	11	Mitochondrial translation
-	70	–	–

## Discussion

Many studies have focused their efforts on identifying the regulatory processes between genes and their potential functions in the GRNs. Examining the gene–gene interaction is one of the useful approaches for these studies. However, in a real biological environment, there are many confounding factors that may influence the gene–gene interaction and make their relationship ambiguous. The MR method is one of the powerful tools to correct these confounding effects in the causality analysis. With various MR models being proposed, IVW MR allows multiple IVs in the model to increase the statistical power of the inference. We introduce a new statistical method referred to as MR-GGI using MR approach to infer causality between genes. We applied one gene as the exposure, the other gene as the outcome, and one or more cis-SNPs for the genes as instrumental variable(s) to infer the interaction between two genes.

Using various simulated datasets, we showed that MR-GGI successfully controls the type 1 error and retains statical power under confounding effects. Furthermore, type I errors are controlled by MR-GGI regardless of the number of IVs, the existence of weak cis-eQTLs, or the confounding effects in the data. In addition, using the DREAM5 dataset, a gold standard dataset for gene network analysis, we compared our method with previous causal inference methods: PC algorithms [17], MRPC [18], and MMHC [19]. Comparing the F1 scores, MR-GGI results in higher scores than other methods, especially when the data contains weak cis-eQTLs, such as in the case of the DREAM5 dataset, where MR-GGI closely maintained its performance, while others failed. Lastly, utilizing the yeast dataset, we constructed yeast GRN using gene–gene interactions estimated by MR-GGI. We obtained a total of 6 clusters, and from gene ontology [23] term analysis, we found clusters C3, C4, and C6 were related to cytoplasmic translation, ribosome biogenesis, and mitochondrial translation, respectively. Additionally, C1, C2, and C5 were related to ATP metabolism, NADP and NADPH metabolism, and regulation process, respectively. Especially mitochondria harbor their own genome, and interestingly, an independent cluster, C6, was related to mitochondrial translation.

There are some limitations in the MR-GGI. It finds gene–gene interactions based on the MR model under the assumption that instrumental variables are only associated with exposure but not with confounding factors and that instrumental variables affect the outcome solely through exposure. Thus, an invalid IV that violates the MR assumptions may lead to a false inference. However, as MR methods are being developed [11–13], they can be applied to MR-GGI to reduce misinterpretation and increase the performance of the method. If these methods are applied in further research, we can more accurately infer the gene–gene interactions. Furthermore, recently, a large number of cis-eQTLs have become available, and we believe that MR-GGI has sufficient potential to uncover underlying biological regulatory processes and GRNs.

## Material and methods

### Inverse-variance weighted Mendelian randomization

MR became popular over the past decade as it accurately infers causality between exposure (i.e., gene) and outcome (i.e., trait) while mitigating the impact of confounding factors by using genetic variants as instrumental variables (IV). Additional file 1: Supplementary Fig. 1 shows a directed acyclic graph (DAG) for a MR, where Z represents an instrumental variable (IV), X represents an exposure, Y represents an outcome, U represents a confounding factor, ${\beta }_{zx}$ represents effect size of Z on X, ${\beta }_{zy}$ represents effect size of Z on Y, and ${\beta }_{xy}$ represents the effect size of X on Y. The MR model is based on three basic assumptions: IV is associated with exposure; IV is independent of confounding factors that affect exposure and outcome; and there is no causal pathway between IV and outcome other than via exposure.

The causality between X and Y can be estimated by 2-stage least squares (2SLS) which involves conducting regression technique in two stages as follows:

Stage1) Perform a regression of the exposure on IV:

$$X|Z={\alpha }_{0}+{\beta }_{zx}Z+{e}_{1}$$

Stage2) Perform a regression of the outcome on fitted values of the regression in previous stage:

$$Y|\widehat{X}={\beta }_{0}+{\beta }_{xy}\widehat{X}+{e}_{2}$$

If the exposure causally influences Y, the direct effect of Z on Y, ${\beta }_{zy}$ is equal to the product of ${\beta }_{zx}$ and ${\beta }_{xy}$ (${\beta }_{zy}={\beta }_{zx}{\beta }_{xy}$). It can be expressed as follows [25]:

$$\beta_{xy} = \frac{{\beta_{zy} }}{{\beta_{zx} }},$$

where ${\beta }_{xy}$ represents the effect size (coefficient) of X on Y, ${\beta }_{zx}$ represents the effect size of Z on X, and ${\beta }_{zy}$ represents the effect size of Z on Y. When more than one IV is used in the model, the causality of the $i$th IV can be described as follows:

$$\hat{\beta }_{{xy_{i} }} = \frac{{\beta_{{zy_{i} }} }}{{\beta zx_{i} }},$$

where ${{\beta }_{xy}}_{i}$, ${{\beta }_{zx}}_{i}$, and ${{\beta }_{zy}}_{i}$ represent coefficient estimates using the $i$th IV.

IVW MR allows multiple IVs, for which it assumes IVs are independent in the model and applies the IVW method [10] to find the causality $\widehat{{\beta }_{xy}}$ and its standard error ($se\left(\widehat{{\beta }_{xy}}\right)$) as follows:

1 $${\widehat{\beta }}_{xy}= \frac{\sum_{k=1}^{n}{{\beta }_{zx}}_{k} {{\beta }_{zy}}_{k} {se\left({{\beta }_{zy}}_{k}\right)}^{-2}}{\sum_{k=1}^{n}{{{{\beta }_{zx}}_{k} \beta }_{zx}}_{k} {se\left({{\beta }_{zy}}_{k}\right)}^{-2}}$$

2 $$se\left({\widehat{\beta }}_{xy}\right)= \sqrt{\frac{1}{\sum_{k=1}^{n}{{{{\beta }_{zx}}_{k} \beta }_{zx}}_{k} {se({{\beta }_{zy}}_{k})}^{-2}}}$$

### MR-based method for inferring gene–gene interaction

MR-GGI identifies gene–gene interactions by inferring causality between two genes using IVW MR. In the model, one gene is used as an exposure, the other gene is used as an outcome, and the causal cis-SNP(s) for a gene are used as IV(s).

First, we select independent cis-SNPs for each gene to use as IVs in the MR model by applying a fine-mapping method to the genotype and expression data. Then, we select candidate gene–gene pairs that have a correlation above a predefined threshold. For all the candidate gene–gene pairs, MR-GGI performs a process for identifying gene–gene interactions based on the MR method. We are testing interaction between two genes, $${g}_{i}$$ and $${g}_{j}$$. The association between these genes has four scenarios: (1) independent ($${g}_{i}$$ $$\perp$$ $${g}_{j}$$); (2) $${g}_{i}$$ affects $${g}_{j}$$ ($${g}_{i}$$ → $${g}_{j}$$); (3) $${g}_{j}$$ affects $${g}_{i}$$($${g}_{i}$$ ← $${g}_{j})$$; and 4) $${g}_{i}$$ and $${g}_{j}$$ affects each other ($${g}_{i}$$ ⇄ $${g}_{j}$$). To test the scenarios, MR-GGI estimates the causal effect of $${g}_{i}$$ on $${g}_{j}$$ ($${\widehat{\beta }}_{{g}_{i}{g}_{j}}$$) by applying MR, incorporating $${g}_{i}$$ as an exposure, $${g}_{j}$$ as an outcome, and cis-SNP(s) for $${g}_{i}$$ as IV(s). Then, MR-GGI estimates the causal effect of $${g}_{j}$$ on $${g}_{i}$$ ($${\widehat{\beta }}_{{g}_{j}{g}_{i}}$$) by applying MR, incorporating $${g}_{j}$$ as an exposure, $${g}_{i}$$ as an outcome, and cis-SNP(s) for $${g}_{j}$$ as IV(s). To find significant associations, the Wald test [26] has been performed (see below for the details). If we find a significant association either in $${g}_{i}$$ → $${g}_{j}$$ or in $${g}_{j}$$ → $${g}_{i}$$, we accept the second or third scenario, respectively. If we find significant associations in both directions, we accept the third scenario, $${g}_{i}$$ ⇄ $${g}_{j}$$ and if we find significant associations in neither of the directions, we accept the first scenario, $${g}_{i}$$ $$\perp$$ $${g}_{j}$$. Additional file 2: Supplementary Fig. 2 shows how we apply MR to find the interaction between $${g}_{i}$$ and $${g}_{j}$$. Here, $${s}_{i}=\{{s}_{i1}, {s}_{i2},\dots ,{s}_{il}\}$$ represents $$l$$ cis-SNPs that are used as IVs for $${g}_{i}$$, $${s}_{j}=\{{s}_{j1}, {s}_{j2},\dots ,{s}_{jp}\}$$ represents $$p$$ cis-SNPs that are used as IVs for $${g}_{j}$$, $${\beta }_{{g}_{i}{g}_{j}}$$ represents effect size of $${g}_{i}$$ on $${g}_{j}$$, $${\beta }_{{s}_{i}{g}_{j}}$$ represents effect size of $${s}_{i}$$ on $${g}_{j}$$, $${\beta }_{{s}_{ik}{g}_{j}}$$ represents effect size of $${s}_{ik}$$ on $${g}_{j}$$, and $$u$$ represents a confounding effect that affects both $${g}_{i}$$ and $${g}_{j}$$. Here, the effect size between a cis-SNP and a gene is called the cis-effect.

The causal effect size of ${g}_{i}$ on ${g}_{j}$ (${\widehat{\beta }}_{{g}_{i}{g}_{j}}$) and ${g}_{j}$ on ${g}_{i}$ (${\widehat{\beta }}_{{g}_{j}{g}_{i}}$) can be estimated from Eqs. (1) and (2) as follows:

3 $${\widehat{\beta }}_{{g}_{i}{g}_{j}}= \frac{\sum_{k=1}^{n}{\beta }_{{s}_{ik}{g}_{i}} {\beta }_{{s}_{ik}{g}_{j}} {se({\beta }_{{s}_{ik}{g}_{j}})}^{-2}}{\sum_{k=1}^{n}{\beta }_{{s}_{ik}{g}_{i}} {\beta }_{{s}_{ik}{g}_{i}} {se({\beta }_{{s}_{ik}{g}_{j}})}^{-2}}$$

4 $${\widehat{\beta }}_{{g}_{j}{g}_{i}}= \frac{\sum_{k=1}^{m}{\beta }_{{s}_{jk}{g}_{j}} {\beta }_{{s}_{jk}{g}_{i}} {se({\beta }_{{s}_{jk}{g}_{i}})}^{-2}}{\sum_{k=1}^{m}{\beta }_{{s}_{jk}{g}_{j}} {\beta }_{{s}_{jk}{g}_{j}} {se({\beta }_{{s}_{jk}{g}_{i}})}^{-2}}$$

Here, $se({\beta }_{{s}_{ik}{g}_{j}})$ represents standard error of ${\beta }_{{s}_{ik}{g}_{j}}$ and $se({\beta }_{{s}_{jk}{g}_{i}})$ represents standard error of ${\beta }_{{s}_{jk}{g}_{i}}$, and they can be estimated as follows:

5 $$se\left({\widehat{\beta }}_{{g}_{i}{g}_{j}}\right)= \sqrt{\frac{1}{\sum_{k=1}^{n}{\beta }_{{s}_{ik}{g}_{i}} {\beta }_{{s}_{ik}{g}_{i}} {se({\beta }_{{s}_{ik}{g}_{j}})}^{-2}}}$$

6 $$se\left({\widehat{\beta }}_{{g}_{j}{g}_{i}}\right)= \sqrt{\frac{1}{\sum_{k=1}^{m}{\beta }_{{s}_{jk}{g}_{j}} {\beta }_{{s}_{jk}{g}_{j}} {se({\beta }_{{s}_{jk}{g}_{i}})}^{-2}}}$$

When inferring the gene–gene interaction, we excluded gene–gene pairs with overlapping cis-SNPs that effect both exposure gene and outcome gene, to satisfy one of the MR assumptions; There is no causal pathway between IV and outcome other than via exposure. From the Wald test, we can calculate *p*-values for causal effect of all the candidate gene pairs, and Bonferroni correction was applied for the multiple testing to adjust *p*-values and identify the significant causal directions.

### MR-GGI generative model

$n$ Is the number of samples, $l$ is the number of cis-SNPs for the $i$th gene, $p$ is the number of cis-SNPs for the $j$th gene. Two genes, ${g}_{i}$ and ${g}_{j}$ were generated based on the following generative model in the simulation studies.

7 $${g}_{i}={S}_{i} {{\beta }_{{s}_{i}{g}_{i}}}^{T}+{u}_{i}+{e}_{i}$$

8 $${g}_{j}={S}_{j} {{\beta }_{{s}_{j}{g}_{j}}}^{T}+{\beta }_{{g}_{i}{g}_{j}} {g}_{i}+{u}_{j}+{e}_{j}$$

9 $$\left(\begin{array}{c}{u}_{i}\\ {u}_{j}\end{array}\right)\sim N\left(0, \left(\begin{array}{cc}1& \rho \\ \rho & 1\end{array}\right)\right)$$

Here, ${g}_{i}$ and ${g}_{j}$ are vectors of length $n$ containing expression values of the $i$th and $j$th genes, respectively. ${S}_{i}$ and ${S}_{j}$ are $n$ x $l$ and $n$ x $p$ matrices, containing sets of cis-SNPs of the $i$th and $j$th genes, respectively. ${\beta }_{{g}_{i}{g}_{j}}$ represents effect size of ${g}_{i}$ on ${g}_{j}$, ${\beta }_{{s}_{i}{g}_{j}}$ represents effect size of ${s}_{i}$ on ${g}_{j}$. ${u}_{i}$ and ${u}_{j}$ are vectors of length $n$, containing confounding effects of ${g}_{i}$ and ${g}_{j}$. Furthermore, ${u}_{i}$ and ${u}_{j}$ follow a multivariate normal distribution with a mean of 0 and a covariance of $$\left(\begin{array}{cc}1& \rho \\ \rho & 1\end{array}\right)$$, where $\rho$ represents the correlation between ${u}_{i}$ and ${u}_{j}$. ${e}_{i}$ and ${e}_{j}$ are residual errors of ${g}_{i}$ and ${g}_{j}$, which follow a normal distribution with a mean of 0 and a variance of 1.

### Simulation studies

For two genes (${g}_{1}$ and ${g}_{2}$), cis-SNPs were sampled from binomial distribution with minor allele frequency (MAF) of 0.3 and were coded additively.

10 $${s}_{ik}\sim Bin\left(2, 0.3\right)$$

11 $${s}_{jk}\sim Bin\left(2, 0.3\right)$$

Here, ${s}_{1k}$ represents the $k$th cis-SNP for ${g}_{1}$ and ${s}_{2k}$ represents the $k$th cis-SNP for ${g}_{2}$. Gene expressions of ${g}_{1}$ with $l$ number of cis-SNPs and ${g}_{2}$ with $p$ number of cis-SNPs were simulated as follows:

12 $${g}_{1}=\left({s}_{11}, {s}_{12}, \dots , {s}_{1l}\right) {\left({\beta }_{{s}_{11}{g}_{1}}, {\beta }_{{s}_{12}{g}_{1}}, \dots , {\beta }_{{s}_{1l}{g}_{1}}\right)}^{T}+{u}_{1}+{e}_{1}$$

13 $${g}_{2}=\left({s}_{21}, {s}_{22}, \dots , {s}_{2p}\right) {\left({\beta }_{{s}_{21}{g}_{2}}, {\beta }_{{s}_{22}{g}_{2}}, \dots , {\beta }_{{s}_{2p}{g}_{2}}\right)}^{T}+{\beta }_{{g}_{1}{g}_{2}} {g}_{1}+{u}_{2}+{e}_{2}$$

Here, ${\beta }_{{s}_{ik}{g}_{j}}$ represents the effect size of ${s}_{ik}$ on ${g}_{i}$, ${\beta }_{{g}_{i}{g}_{j}}$ represents the effect size of ${g}_{i}$ on ${g}_{j}$, and ${e}_{i}$ represents the residual error of ${g}_{i}$ that follows a normal distribution of ${e}_{1}\sim N\left(0, 1\right)$. ${u}_{i}$ and ${u}_{j}$ represent the confounding effect of ${g}_{i}$ and ${g}_{j}$, respectively, which follows a multivariate normal distribution with correlation ($\rho$); $$\left(\begin{array}{c}{u}_{1}\\ {u}_{2}\end{array}\right)\sim MVN\left(0, \left(\begin{array}{cc}1& \rho \\ \rho & 1\end{array}\right)\right)$$.

To show that MR-GGI controls the type I error in various scenarios, we simulated various datasets, giving ${\beta }_{{g}_{1}{g}_{2}}=0$. First, to show that MR-GGI controls the false positives in cases of different numbers of IVs, we simulated 3 sets of 10,000 datasets with two genes, where 1, 3, or 5 cis-SNPs were simulated for each set as follows:

14 $${g}_{1}=\left({s}_{11}, {s}_{12}, \dots , {s}_{1l}\right) {\left({\beta }_{{s}_{11}{g}_{1}}, {\beta }_{{s}_{12}{g}_{1}}, \dots , {\beta }_{{s}_{1l}{g}_{1}}\right)}^{T}+{e}_{1}$$

15 $${g}_{2}=\left({s}_{21}, {s}_{22}, \dots , {s}_{2p}\right) {\left({\beta }_{{s}_{21}{g}_{2}}, {\beta }_{{s}_{22}{g}_{2}}, \dots , {\beta }_{{s}_{2p}{g}_{2}}\right)}^{T}+{e}_{2}$$

For 1 cis-SNP case, ${\beta }_{{s}_{11}{g}_{1}}={\beta }_{{s}_{21}{g}_{2}}=0.6$, for 3 cis-SNPs case, $\left\{{\beta }_{{s}_{11}{g}_{1}},{\beta }_{{s}_{12}{g}_{1}},{\beta }_{{s}_{13}{g}_{1}}\right\}=\left\{{\beta }_{{s}_{21}{g}_{2}},{\beta }_{{s}_{22}{g}_{2}},{\beta }_{{s}_{23}{g}_{2}}\right\}=\{0.55, 0.4, 0.25\}$, and for 5 cis-SNP case $\left\{{\beta }_{{s}_{11}{g}_{1}},{\beta }_{{s}_{12}{g}_{1}},{\beta }_{{s}_{13}{g}_{1}},{\beta }_{{s}_{14}{g}_{1}},{\beta }_{{s}_{15}{g}_{1}}\right\}=\left\{{\beta }_{{s}_{21}{g}_{2}},{\beta }_{{s}_{22}{g}_{2}},{\beta }_{{s}_{23}{g}_{2}},{\beta }_{{s}_{24}{g}_{2}},{\beta }_{{s}_{25}{g}_{2}}\right\}=\left\{0.55, 0.4, 0.35, 0.3, 0.25\right\}$ were used to generate datasets.

Second, to simulate datasets with weak IVs, we simulated 3 sets of 10,000 datasets of two genes, and 3 cis-SNPs were simulated for each set. For this experiment, the datasets contain 1, 2, or 3 weak IVs out of 3 IVs, for which a relatively small effect size of 0.1 was used, following a previous study [16]. For 1 weak IV case, $\left\{{\beta }_{{s}_{11}{g}_{1}},{\beta }_{{s}_{12}{g}_{1}},{\beta }_{{s}_{13}{g}_{1}}\right\}=\left\{{\beta }_{{s}_{21}{g}_{2}},{\beta }_{{s}_{22}{g}_{2}},{\beta }_{{s}_{23}{g}_{2}}\right\}=\{0.4, 0.3, 0.1\}$, for 2 weak IVs case, $\left\{{\beta }_{{s}_{11}{g}_{1}},{\beta }_{{s}_{12}{g}_{1}},{\beta }_{{s}_{13}{g}_{1}}\right\}=\left\{{\beta }_{{s}_{21}{g}_{2}},{\beta }_{{s}_{22}{g}_{2}},{\beta }_{{s}_{23}{g}_{2}}\right\}=\left\{0.4, 0.1, 0.1\right\}$, and for 3 weak IVs case, $\left\{{\beta }_{{s}_{11}{g}_{1}},{\beta }_{{s}_{12}{g}_{1}},{\beta }_{{s}_{13}{g}_{1}}\right\}=\left\{{\beta }_{{s}_{21}{g}_{2}},{\beta }_{{s}_{22}{g}_{2}},{\beta }_{{s}_{23}{g}_{2}}\right\}=\left\{0.1, 0.1, 0.1\right\}$ were used to generate the datasets.

Third, to show that MR-GGI successfully controls false positives under confounding effects, we simulated 7 sets of 10,000 datasets with 2 genes, where 3 cis-SNPs were simulated for each set as follows:

16 $${g}_{1}=\left({s}_{11}, {s}_{12},{s}_{13}\right) {\left({\beta }_{{s}_{11}{g}_{1}}, {\beta }_{{s}_{12}{g}_{1}},{\beta }_{{s}_{13}{g}_{1}} \right)}^{T}+{u}_{1}+{e}_{1}$$

17 $${g}_{2}=\left({s}_{21}, {s}_{22}, {s}_{23}\right) {\left({\beta }_{{s}_{21}{g}_{2}}, {\beta }_{{s}_{22}{g}_{2}},{\beta }_{{s}_{23}{g}_{2}} \right)}^{T}+{u}_{2}+{e}_{2}$$

Here, $\left\{{\beta }_{{s}_{11}{g}_{1}}, {\beta }_{{s}_{12}{g}_{1}},{\beta }_{{s}_{13}{g}_{1}}\right\}=\left\{{\beta }_{{s}_{21}{g}_{2}}, {\beta }_{{s}_{22}{g}_{2}},{\beta }_{{s}_{23}{g}_{2}}\right\}=\left\{0.55, 0.4, 0.25\right\}$ and correlation between ${u}_{1}$ and ${u}_{2}$ ($\rho$) of [− 0.9, − 0.5, − 0.2, 0, 0.2, 0.5, 0.9] were simulated for each dataset.

Lastly, to show that MR-GGI retains statistical power under confounding effects, we simulated 21 sets of 1000 datasets with 2 genes, and for each gene, 3 cis-SNPs were simulated with confounding effects as follows:

18 $${g}_{1}=\left({s}_{11}, {s}_{12}, {s}_{13}\right) {\left({\beta }_{{s}_{11}{g}_{1}}, {\beta }_{{s}_{12}{g}_{1}},{\beta }_{{s}_{13}{g}_{1}}\right)}^{T}+{u}_{1}+{e}_{1}$$

19 $${g}_{2}=\left({s}_{21}, {s}_{22}, {s}_{23}\right) {\left({\beta }_{{s}_{21}{g}_{2}}, {\beta }_{{s}_{22}{g}_{2}},{\beta }_{{s}_{23}{g}_{2}}\right)}^{T}+{\beta }_{{g}_{1}{g}_{2}} {g}_{1}+{u}_{2}+{e}_{2}$$

Here, $\left\{{\beta }_{{s}_{11}{g}_{1}}, {\beta }_{{s}_{12}{g}_{1}},{\beta }_{{s}_{13}{g}_{1}}\right\}=\left\{{\beta }_{{s}_{21}{g}_{2}}, {\beta }_{{s}_{22}{g}_{2}},{\beta }_{{s}_{23}{g}_{2}}\right\}=\left\{0.55, 0.4, 0.25\right\}$, ${\beta }_{{g}_{1}{g}_{2}}$ of [− 0.7, − 0.5, − 0.3, − 0.15, − 0.05, − 0.025, 0, 0.025, 0.05, 0.15, 0.3, 0.5, 0.7], and correlation between ${u}_{1}$ and ${u}_{2}$ ($\rho$) of [− 0.9, − 0.5, − 0.2, 0, 0.2, 0.5, 0.9] were used for each dataset.

In addition, we changed the number of IVs to show how the power changes with the number of IVs. For this experiment, the datasets were simulated as follows:

20 $${g}_{1}=\left({s}_{11}, {s}_{12},{s}_{13},{s}_{14},{s}_{15}\right){\left({\beta }_{{s}_{11}{g}_{1}},{\beta }_{{s}_{12}{g}_{1}},{\beta }_{{s}_{13}{g}_{1}},{\beta }_{{s}_{14}{g}_{1}},{\beta }_{{s}_{15}{g}_{1}}\right)}^{T}+{e}_{1}$$

21 $${g}_{1}=\left({s}_{21}, {s}_{22},{s}_{23},{s}_{24},{s}_{25}\right){\left({\beta }_{{s}_{21}{g}_{1}},{\beta }_{{s}_{22}{g}_{1}},{\beta }_{{s}_{23}{g}_{1}},{\beta }_{{s}_{24}{g}_{1}},{\beta }_{{s}_{25}{g}_{1}}\right)}^{T}+{\beta }_{{g}_{1}{g}_{2}}{g}_{1}+{e}_{2}$$

where, $\left\{{\beta }_{{s}_{11}{g}_{1}}, {\beta }_{{s}_{12}{g}_{1}},{\beta }_{{s}_{13}{g}_{1}}, {\beta }_{{s}_{14}{g}_{1}},{\beta }_{{s}_{15}{g}_{1}}\right\}=\left\{{\beta }_{{s}_{21}{g}_{2}}, {\beta }_{{s}_{22}{g}_{2}},{\beta }_{{s}_{23}{g}_{2}},{\beta }_{{s}_{24}{g}_{2}},{\beta }_{{s}_{25}{g}_{2}}\right\}$=$\left\{0.55, 0.4,\mathrm{ 0.35,0.3,0.25}\right\}$, and ${\beta }_{{g}_{1}{g}_{2}}$ of [− 0.7, − 0.5, − 0.3, − 0.15, − 0.05, − 0.025, 0, 0.025, 0.05, 0.15, 0.3, 0.5, 0.7] were used. We simulated 1,000 datasets for each set. This test consisted of three cases where the top 1, 3, and 5 cis-SNPs were used as IVs in order of increasing cis-effect.

### DREAM5 dataset analysis

We compared MR-GGI with other network construction methods using DREAM5 [14] (https://www.synapse.org/#!Synapse:syn2820440/files/). DREAM5 comprises 15 simulated datasets created for the 2010 DREAM5 Systems Genetics In-silico Network subchallenge, each incorporating various scales of Recombinant Inbred Lines (RILs) [27], utilized for inferring gene networks. This dataset provides simulated genotype and expression data for synthetic gene regulatory networks. The DREAM5 sub-datasets containing 1000 genes consisted of 100, 300, and 999 samples from 5 different networks each. Each gene has exactly one corresponding genotype value in every 15 sub-datasets. In every sub-dataset, each gene has exactly one corresponding genotype variable, and the gold standard (correct edges) was obtained.

We transform the genotype data and the expression data for each gene to have a mean of 0 and a variance of 1. Gene–gene pairs were selected only up to absolute gene expression correlations of 0.5. To avoid using weak IV, we filtered out the rest of the gene–gene pairs where the absolute cis-effect was less than 0.2. Then, compared with the previous case, we didn’t perform cis-effect filtering to confirm the performance when including weak IV in gene–gene interaction inference.

We used the F1-score to compare the performance of MR-GGI and other methods. The F1-score is calculated as the harmonic mean of precision and recall [28], and it is a proper metric for evaluating model performance in imbalanced datasets.

$$F1=\frac{2\times precision\times recall}{precision+recall}$$

### Yeast dataset analysis

We analyzed yeast datasets to investigate whether MR-GGI performs well not only in simulated data and virtual networks but also in real biological environments. The yeast dataset contains 5,720 genes and 42,052 SNPs in 1,012 yeast segregants from a cross between the BY4617 (BY) strain and the vineyard RM11-1a (RM) strain [15]. Selecting the *cis*-SNP to be used as IV was performed as follows. First, *cis*-eQTLs were identified within $\pm 1$ Mb of each transcription start site (TSS) of a gene. And, we perform fine-mapping with the susie function in the susieR package [16, 17] to select true causal variants based on fine-mapping. Gene–gene pairs were selected only up to absolute gene expression correlations of 0.75.

After inferring gene–gene interactions, we extracted the information about nodes corresponding to genes and edges to construct the yeast GRN. Next, we applied the Louvain algorithm in the igraph R package [22] for clustering. To identify the key roles of each cluster, we performed 2-step functional enrichment analysis using yeast GO biological processes using the GO Term Finder in the Saccharomyces Genome Database (SGD) [23]. First, we obtained the GO terms of all genes for each cluster, and filtered out low centrality (degree centrality < 3) genes in annotated genes. Degree centrality of all genes for each cluster was calculated by using the tidygraph R package [29]. In the second step, we acquired the ‘final GO terms’ for these filtered gene sets to discover key role for each cluster. We used the visNetwork R package [30] for yeast GRN visualization.

### Supplementary Information

**Additional file 1**. Figure 1. A directed acyclic graph for Mendelian randomization.

**Additional file 2**. Figure 2. Association test process of MR-GGI.

**Additional file 3**. Data 1. Biological process of Gene Ontology terms for cluster 1 in yeast GRN

**Additional file 4**. Data 2. Biological process of Gene Ontology terms for cluster 2 in yeast GRN

**Additional file 5**. Data 3. Biological process of Gene Ontology terms for cluster 3 in yeast GRN

**Additional file 6**. Data 4. Biological process of Gene Ontology terms for cluster 4 in yeast GRN

**Additional file 7**. Data 5. Biological process of Gene Ontology terms for cluster 5 in yeast GRN

**Additional file 8**. Data 6. Biological process of Gene Ontology terms for cluster 6 in yeast GRN

## Data Availability

The datasets of DREAM5 are available at http://www.synapse.org/#!Synapse:syn2787209/files/. MR-GGI are downloaded as the MRggi R package (10.5281/zenodo.10108231) on the GitHub platform: https://github.com/hiows/MRggi.
