# Supplementary figures and images for: MR-GGI: accurate inference of gene–gene interactions using Mendelian randomization
Source: BMC Bioinformatics. 2024 May 15;25:192. doi: 10.1186/s12859-024-05808-4 (PMC11094870; doi:10.1186/s12859-024-05808-4)

**
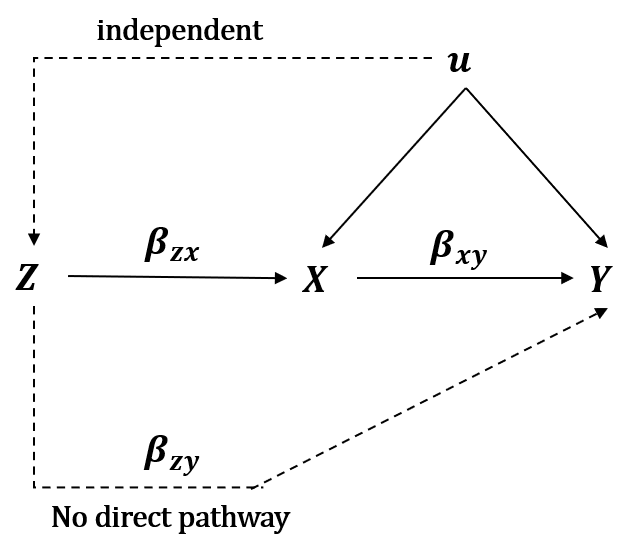
**

**Supplementary Fig. 1** A directed acyclic graph for Mendelian randomization.

Supplement: Supplementary file 1 — Additional file 1. Figure 1. A directed acyclic graph for Mendelian randomization. [file 12859_2024_5808_MOESM1_ESM.docx]

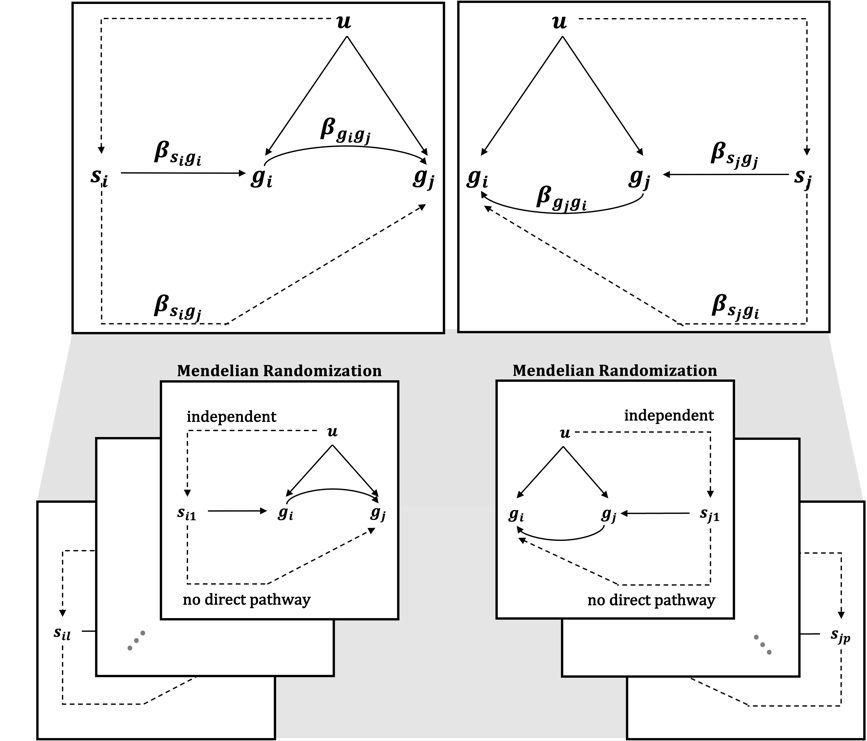


**Supplementary Fig. 2** Association test process of MR-GGI.

Supplement: Supplementary file 2 — Additional file 2. Figure 2. Association test process of MR-GGI. [file 12859_2024_5808_MOESM2_ESM.docx]
